# Environment, lifestyle, and cancer in women

**DOI:** 10.1002/ijgo.70156

**Published:** 2025-04-25

**Authors:** Sara Farina, Alessandra Sabatelli, Stefania Boccia, Giovanni Scambia

**Affiliations:** ^1^ Section of Hygiene, University Department of Life Sciences and Public Health Università Cattolica del Sacro Cuore Rome Italy; ^2^ Department of Woman and Child Health and Public Health‐Public Health Area Fondazione Policlinico Universitario A. Gemelli IRCCS Rome Italy

**Keywords:** cancer risk factors, diet and cancer, environmental exposures, epidemiology of cancer, hormonal influences, Lifestyle and cancer, reproductive factors

## Abstract

Environmental and lifestyle factors significantly contribute to gynecological cancers. The risk of ovarian cancer, one the most lethal gynecological cancer, is associated with obesity, poor dietary habits, and environmental pollutants, exacerbating hormonal imbalances, inflammation, and oxidative stress. Protective factors, such as the Mediterranean diet and oral contraceptives, modulate risk by reducing ovulatory cycles, particularly in genetically predisposed women. Uterine cancer is associated with metabolic factors, with obesity driving hormonal disruptions and systemic inflammation. Physical inactivity and diets rich in animal fats increase the risk of endometrial cancer, along with air pollution and microbiome imbalances contribute to endometrial carcinogenesis. Cervical cancer is primarily driven by persistent high‐risk HPV infection, with smoking enhancing viral persistence and oncogenesis. Nutritional deficiencies in antioxidants and folate weaken immune defenses, while vaginal and gut microbiome dysbiosis fosters neoplastic progression. Vulvar and vaginal cancers, though less common, share risk factors such as obesity, smoking, and occupational exposures, disrupting immune responses and epithelial integrity. Microbial imbalances exacerbate these malignancies, creating a pro‐inflammatory microenvironment. The interplay between modifiable factors and genetic predisposition, including high‐penetrance mutations and polygenic risk scores, highlights the complexity complexity of prevention of gynecological cancers. Epigenetic mechanisms, such as DNA methylation and histone modifications, further modulate susceptibility and tumor progression, influenced by environmental and lifestyle exposures. In addition, promoting and supporting healthy lifestyle changes, including smoking cessation, increased physical activity, and a balanced diet, are crucial for improving long‐term outcomes and quality of life in gynecological cancer survivors. Addressing these factors through personalized prevention, leveraging predictive models incorporating genetics and modifiable risks, enables tailored lifestyle interventions and avoidance of environmental exposures. Combined with equitable public health initiatives, these strategies have the potential to reduce the burden of gynecological cancers and improve women's health globally.

## 1 INTRODUCTION

**FIGURE 1:**
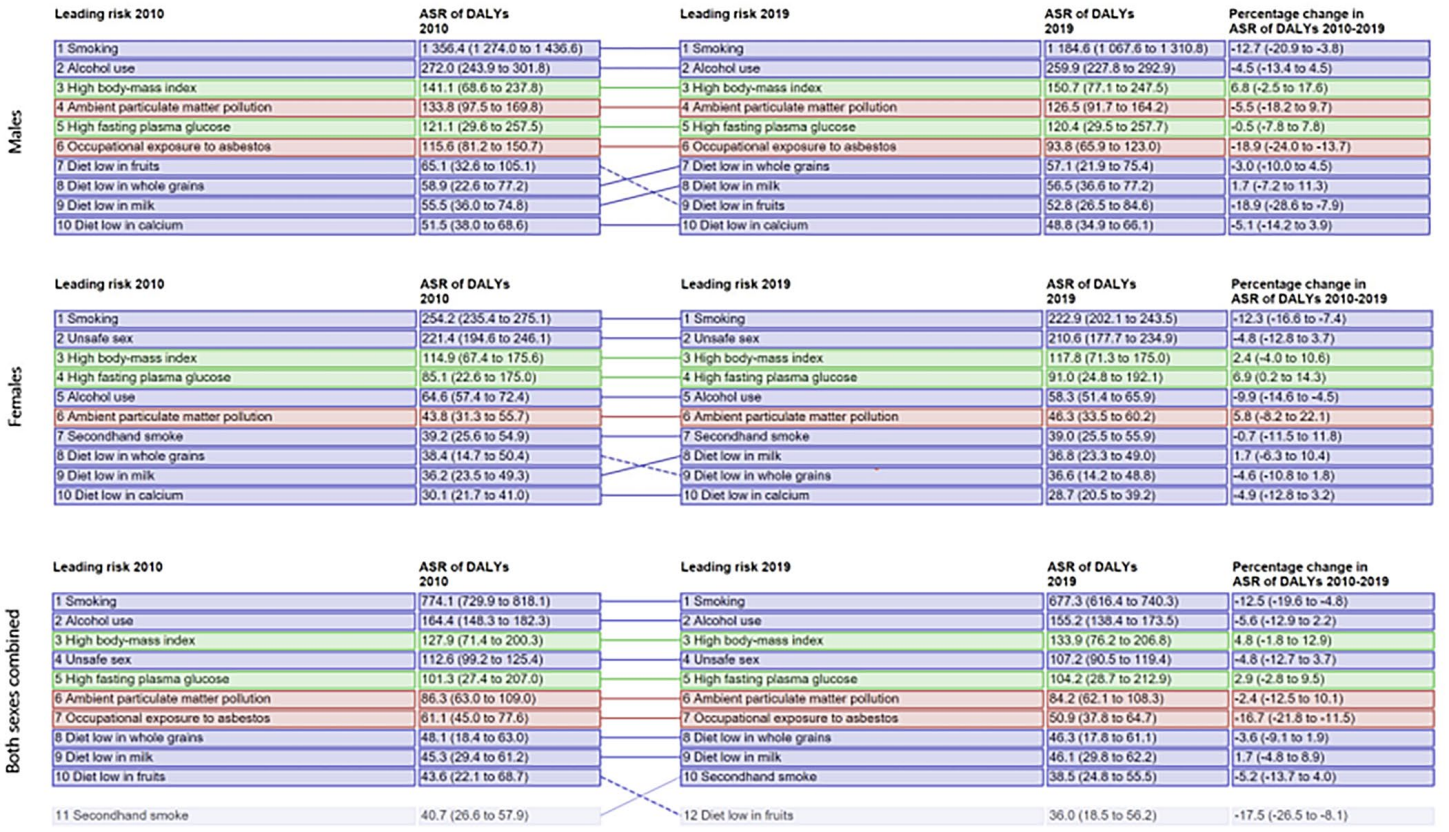
Leading risk factors at the most detailed level for attributable cancer age‐standardized DALY rates, 2010–2019 for the global level, for men, women, and both sexes combined.^4^ The data in parentheses are 95% uncertainty intervals (UIs). Rows are color‐coded as follows: Red = environmental and occupational risk factors; blue = behavioral risk factors; green = metabolic risk factors. Dashed lines indicate a decrease in rank; solid lines indicate an increase or no change in rank. Risk factors at the most detailed level reflect the GBD hierarchy in which these categories of risks fall, ranging from levels 2 to 4. ASR, age‐standardized rate; DALY, disability‐adjusted life‐year; SDI, sociodemographic index.^4^

The impact of environmental exposures and lifestyle behaviors on cancer development has garnered significant attention in recent years. Environmental and lifestyle factors, as defined by the International Agency for Research on Cancer (IARC), refer to exposures to potentially carcinogenic agents found in various environmental media, including soil, water, air, and food.^1^ These exposures, collectively framed within the concept of external exposome, occur across different settings, such as workplaces, homes, and the broader environment, and encompass diverse elements, including all forms of radiation—ionizing, non‐ionizing, and optical—as well as behaviors and habits shaped by individual choices and influenced by socioeconomic and cultural conditions.^1^, ^2^ It has been highlighted that these factors contribute significantly to the global cancer burden, accounting for nearly 50% of all cases. Moreover, they are thought to contribute to a large portion of the remaining 50%, for which causes—apart from genetic predispositions—remain unidentified.^1^ Given the unique biological, hormonal, and socioeconomic factors influencing women's health, these environmental and lifestyle exposures are particularly relevant in the context of female cancers, where they play a critical role in shaping risk profiles and disease outcomes.^3^ Thus, as highlighted by an analysis conducted by the Global Burden of Disease Study, the leading risk factors for cancer‐related disability‐adjusted life years (DALYs) among women globally have shown notable trends between 2010 and 2019.^4^ Smoking and unsafe sex remained the top contributors to the female cancer burden, although their impact declined. Conversely, metabolic risks such as high body mass index (BMI, calculated as weight in kilograms divided by the square of height in meters) showed a slight increase, while environmental factors such as ambient particulate matter pollution and dietary patterns, including diets low in milk or whole grains, also demonstrated increases in their contribution to the cancer burden (Figure 1).

Since many of these factors are modifiable, understanding and addressing them is pivotal for effective primary cancer prevention. Limiting exposure to these risks could substantially reduce their impact. In addition, considering the interplay between environment, lifestyle, and genetic predisposition can precisely predict the individual risk of developing cancer. Understanding and addressing these modifiable factors is pivotal not only for reducing the overall cancer burden but also for mitigating their impact on individuals with a genetic predisposition, as these environmental and lifestyle exposures can influence the manifestation and progression of cancer even in genetically susceptible populations.^3^ Furthermore, environmental and lifestyle factors are closely linked to social inequalities in cancer outcomes, disproportionately affecting the most vulnerable populations. The intricate interplay of these factors underscores the necessity of sustained research to develop strategies that mitigate their influence and promote equitable cancer prevention measures.^3^

This review focuses on the impact of environmental exposures and lifestyle behaviors on cancer in women, exploring the intricate interplay between modifiable and non‐modifiable risk factors. By synthesizing the latest evidence, it aims to elucidate the critical role of risk factors in shaping gynecological cancer risk and progression, while highlighting opportunities for targeted preventive strategies and public health interventions.

## 2 MODIFIABLE RISK FACTORS IN GYNECOLOGICAL CANCER

The sections below provide a comprehensive analysis of how environmental and lifestyle factors influence the development and progression of gynecological cancers, as summarized in Table 1. The levels of association reported in the table where derived form the literature included in the review.^6^, ^7^, ^8^, ^9^, ^10^, ^11^, ^12^, ^13^, ^14^, ^15^, ^16^, ^17^, ^18^, ^19^, ^20^, ^21^, ^22^

**TABLE 1:** Summary of modifiable risk factor and their potential level of association on gynecological cancers.

Risk factors	Ovary	Corpus uteri	Cervix	Vulva/vagina
Obesity (BMI ≥30)	High	High	Moderate	High
Dietary habits (high animal fat, low fiber)	High	High	Moderate	Moderate
Physical inactivity	Moderate	High	Low	Moderate
HPV infection	N/A	N/A	High	High
Smoking (active/passive)	Moderate	N/A	High	High
Air pollution (PM2.5, CO, SO2)	Moderate	Moderate	Low	Low
Occupational exposure (e.g. asbestos, PAHs, pesticides)	High	N/A	Low	High
Microbiome imbalance	Moderate	Moderate	High	Moderate
Reproductive factors (e.g. parity, contraceptives)	High	Low	Low	Low

*Note*: The table categorizes the impact of various modifiable risk factors on gynecological cancers, including ovarian, uterine, cervical, and vulvar/vaginal cancers. The levels of association are defined as follows: “High” indicates a convincing increased cancer risk; “Moderate” reflects a probable increased risk; “Low” denotes a limited or suggestive increased risk; and “N/A” signifies no notable impact or insufficient evidence of association.

### 2.1 Ovarian cancer

Ovarian cancer is one of the most lethal gynecological malignancies, with an incidence of more than 320 000 cases worldwide and over 200 000 deaths reported in 2022.^5^ It is influenced by a combination of modifiable lifestyle factors, genetic predisposition, and environmental exposures. High BMI is a particularly impactful risk factor, with obesity contributing significantly to the disease's development. According to the Global Burden of Disease Study, there has been a 7.9% rise in ovarian cancer‐related deaths attributed to high BMI in recent decades.^6^ Women with a BMI ≥30 have a substantially higher risk compared to those with a BMI <22.5, with the risk increasing progressively with higher BMI levels.^7^ Obesity exacerbates ovarian cancer risk through hormonal mechanisms, including excessive estrogen production by adipose tissue, as well as increased levels of insulin‐like growth factors, which promote cellular proliferation and survival. Furthermore, chronic low‐grade inflammation linked to obesity creates a microenvironment conducive to tumor progression.^6^, ^7^, ^8^, ^9^

Dietary habits also play a crucial role in ovarian cancer risk. Diets high in animal fats and low in fruits, vegetables, and phytoestrogens have been associated with increased risk. In contrast, adherence to a Mediterranean diet, rich in plant‐based foods, antioxidants, and healthy fats, has shown protective effects. Specific dietary components, such as isoflavones and flavonoids, are thought to influence cancer risk by modulating estrogen receptor activity and reducing oxidative stress.^7^, ^8^, ^9^

Reproductive factors interconnect with these modifiable risks, as multiparity and long‐term use of oral contraceptives are known to reduce ovarian cancer risk by decreasing the lifetime number of ovulatory cycles. This reduction in ovulation‐associated inflammation and oxidative stress may be particularly protective in women carrying *BRCA1* and *BRCA2* mutations.^6^, ^7^, ^8^, ^9^

Environmental exposures add another layer of complexity. Air pollution, including exposure to particulate matter (PM) 2.5, CO, and SO2, has been linked to increased ovarian cancer risk.^10^ Studies demonstrate that pollutants can cause oxidative stress and systemic inflammation, mechanisms that may further compromise the DNA repair pathways in individuals with genetic susceptibilities like *BRCA* mutations.^10^ Similarly, occupational exposure to asbestos, a known carcinogen, compounds genetic risks, particularly in individuals already predisposed to tumorigenesis due to inherited mutations.^11^

### 2.2 Corpus uteri cancer

Uterine cancer, particularly endometrial cancer, is the most prevalent gynecological malignancy in high‐income countries, with over 420 000 cases and nearly 100 000 deaths globally reported in 2022.^5^ Its incidence is strongly associated with metabolic and hormonal factors, primarily obesity, which is the leading risk factor. Women with a BMI >30 face a two‐ to threefold increased risk compared to those with a BMI <22.5. This elevated risk is linked to hormonal imbalances driven by excess adipose tissue, which increases estrogen levels and fosters endometrial changes.^7^, ^8^, ^12^

Physical inactivity further contributes by promoting metabolic syndrome and insulin resistance, which are associated with heightened cancer susceptibility. Dietary patterns also play a crucial role: plant‐based diets have demonstrated protective benefits, while high consumption of animal fats and low dietary fiber increase the risk.^7^, ^8^

Environmental exposures, such as air pollution—specifically PM2.5—and chemical agents—particularly pesticides—have been implicated in uterine cancer due to their association with systemic inflammation and hormonal disruptions.^10^, ^12^, ^13^, ^14^ In addition, recent evidence suggests that alterations in the endometrial microbiome, characterized by reduced diversity and increased pathogenic bacteria, may increase cancer risk through inflammatory mechanisms.^15^, ^16^

### 2.3 Cervical cancer

Cervical cancer continues to represent a substantial global health burden, with more than 660 000 cases and an estimated 350 000 deaths reported in 2022, highlighting its pervasive impact on women's health worldwide.^5^ The primary etiological factor driving this malignancy is the persistent infection with HPV, which plays a critical role in the pathogenesis of cervical cancer. However, the mere presence of HPV is insufficient to explain the development of the disease in all cases.^7^, ^8^, ^17^ A growing body of evidence underscores the significant influence of environmental and lifestyle factors in modulating the risk and progression of cervical cancer, shaping its incidence globally.^17^

Behavioral factors, particularly those related to sexual practices, are key contributors to cervical cancer risk.^17^ Early sexual debut and multiple sexual partners are strongly associated with an increased likelihood of HPV exposure, as these behaviors facilitate the transmission of the virus. Smoking is another well‐documented risk factor that exacerbates the persistence of HPV infection.^17^, ^18^ Tobacco exposure not only doubles the risk of HPV persistence but also increases the probability of its progression to malignancy. This effect is not limited to active smokers; passive smoking has been linked to an approximately 20% higher risk of developing cervical cancer, emphasizing the profound impact of tobacco‐related exposures even in non‐smokers.^8^, ^17^

In addition to behavioral factors, nutritional deficiencies represent another critical dimension of risk. A lack of essential micronutrients, such as folate and antioxidants, weakens immune defenses and compromises DNA repair mechanisms, thereby fostering an environment conducive to HPV persistence and neoplastic transformation. These findings suggest that dietary interventions aimed at enhancing nutrient intake could play a protective role in mitigating cervical cancer risk.^8^

The role of the vaginal microbiome has emerged as a pivotal factor in the progression of cervical cancer. A Lactobacillus‐dominated vaginal microbiota is associated with a protective effect, as these bacteria contribute to maintaining an acidic vaginal pH and inhibit the growth of pathogenic microorganisms.^15^ In contrast, bacterial vaginosis, characterized by microbial imbalance, is linked to increased HPV persistence and progression to cervical intraepithelial neoplasia and cancer.^19^, ^20^ Furthermore, emerging evidence suggests that poor oral hygiene and oral health may also contribute to HPV persistence, potentially due to sexual behaviors. Restoration of vaginal microbiota through the use of probiotics has shown promise as a potential strategy for reducing the risks associated with HPV persistence and cervical neoplasia.^15^, ^16^, ^19^ In addition, alterations in the gut microbiome also play a significant role in cervical cancer, through its impact on immune modulation, inflammation, and metabolic processes. In this context, neuroendocrine signaling between the gut microbiome, brain, and immune system influences inflammatory responses, metabolic disorders, and tumor progression.^16^ This gut‐brain‐immune axis can modulate systemic inflammation and immune responses, potentially affecting cancer development and progression. Chronic stress, anxiety, and depression can alter the gut microbiome composition, leading to dysbiosis. This dysregulation can impair immune responses and increase susceptibility to cancer by promoting a pro‐inflammatory state and altering metabolic pathways.^16^

### 2.4 Vulvar and vaginal cancers

Vaginal cancer is less common, with approximately 20 000 cases worldwide in 2022, but its impact is still significant, with an estimated 8200 deaths.^5^ Similarly, vulvar cancer accounts for almost 50 000 cases globally, with approximately 18 500 deaths reported in the same year.^5^

Although their incidence is lower compared to other gynecological cancers, vulvar and vaginal cancers are still strongly influenced by environmental and lifestyle factors. Obesity is a significant risk factor for vulvar and vaginal cancers, primarily due to its association with chronic inflammation, hormonal imbalances, and metabolic dysregulation.^21^, ^22^ These mechanisms are specifically relevant for vulvar squamous cell carcinomas, with obesity being strongly associated with invasive forms of the disease.^21^

Smoking, particularly in HPV‐associated cases, is a significant risk factor, impairing local immune defenses and facilitating viral persistence.^21^, ^22^ Occupational exposure to carcinogens such as polycyclic aromatic hydrocarbons (PAHs) further increases risk by inducing chronic inflammation and DNA damage.^10^, ^13^

Preliminary evidence suggests that both the vulvar, vaginal, and gut microbiome may also contribute to cancer development, influencing local immune responses and epithelial integrity.^15^, ^16^ Microbial imbalances are thought to promote localized inflammation, creating a microenvironment conducive to carcinogenesis.^15^, ^16^ Furthermore, dysbiosis may compromise the immune system's ability to clear oncogenic infections such as HPV, allowing these viruses to persist and increase the risk of malignant transformation.^15^, ^16^

## 3 INTERPLAY BETWEEN MODIFIABLE AND NON‐MODIFIABLE RISK FACTORS IN GYNECOLOGICAL CANCER

The development of gynecological cancers is driven by a complex interaction between genetic and environmental factors.^7^, ^8^ Among non‐modifiable risk factors, high‐penetrance germline mutations, such as those in *BRCA1* and *BRCA2*, are particularly significant. These mutations confer lifetime ovarian cancer risks of approximately 39%–44% and 11%–17%, respectively, and are also associated with increased risks of breast, fallopian tube, and peritoneal cancers.^23^ Similarly, Lynch syndrome, caused by mutations in mismatch repair (MMR) genes such as *MLH1*, *MSH2*, *MSH6*, and *PMS2*, elevates the risk of endometrial cancer (up to 60%) and ovarian cancer (3%–24%).^24^ Other hereditary syndromes, such as Peutz–Jeghers syndrome (mutations in *STK11*) and Cowden syndrome (mutations in *PTEN*), contribute to gynecological cancer risk by disrupting tumor suppressor pathways and mechanisms involved in genomic stability.^24^

The interaction between these high‐penetrance mutations and environmental or lifestyle factors is fundamental in modulating individual cancer risks. Obesity, for example, exacerbates the oncogenic potential of *BRCA1* and *BRCA2* mutations by increasing systemic inflammation, hormonal imbalances, and estrogen levels derived from adipose tissue, creating an environment that promotes tumor development.^25^ Conversely, protective interventions, such as dietary modifications and regular physical activity, may help counteract some of the negative impacts by reducing chronic inflammation and stabilizing hormonal pathways. The use of oral contraceptives has also been shown to lower ovarian cancer risk, even in *BRCA* mutation carriers, though its effectiveness can vary depending on genetic background.^25^

Beyond high‐penetrance mutations, polygenic risk scores (PRSs) have emerged as a valuable tool to estimate an individual's genetic predisposition to several diseases, including gynecological cancers. PRSs aggregate the effects of numerous common genetic variants identified through genome‐wide association studies (GWAS), each contributing to a small effect, to provide a cumulative risk score.^26^ These scores are calculated by summing the weighted effects of multiple genetic variants, where each variant's weight is determined by its association with the disease in GWAS, allowing individuals to be stratified into different risk categories based on their PRS distribution.^20^ For example, women in the highest tertile of PRS for endometrial cancer exhibit a 2.1‐fold increased risk compared to those in the lowest tertile.^27^ Similarly, studies have shown that women in the top fifth percentile of the PRS for ovarian cancer have a 3.4‐fold increased risk compared to those in the bottom 5%, demonstrating that PRS is a strong predictor of disease.^28^ PRSs are particularly useful for individuals without high‐penetrance mutations but with a familial history of cancer, particularly when integrated with factors such as age, BMI, and reproductive history.^26^ Moreover, PRSs can refine our understanding of gene–environment interactions. For example, lifestyle factors, such as smoking, obesity, or hormone use, can have differential effects on cancer risk depending on an individual's genetic predisposition as determined by their PRS.^26^ For instance, a study conducted by Pearce et al. highlighted the combined and interactive effects of environmental and genetic risk factors, including PRS, in ovarian cancer, emphasizing the importance of considering both types of factors in risk models.^23^ Thus, the CanRisk model is designed to predict the risk of breast and ovarian cancer, integrating multiple factors, including family history, rare pathogenic variants in cancer susceptibility genes, PRSs, and lifestyle, hormonal, and clinical features. This approach allows for more precise risk stratification and personalized prevention strategies, including lifestyle changes and medical preventive interventions, ultimately improving patient outcomes.^29^

Epigenetics provides additional insights into gynecological cancers, revealing how gene expression is regulated by external factors, including environment, smoking, and diet, without altering DNA sequences.^30^ Aberrant DNA methylation is a key feature in cancers like ovarian, endometrial, and cervical. For example, hypermethylation of tumor suppressor genes such as *PTEN* and *MLH1* in endometrial cancer inactivates regulatory pathways, while in ovarian cancer, abnormal *BRCA1* methylation impairs DNA repair, contributing to tumor progression. Hypomethylation, conversely, promotes genomic instability by activating oncogenes or transposable elements.^30^

Histone modifications also play a significant role in gynecological cancers. The altered histone acetylation and methylation affect chromatin structure and gene expression. For instance, in ovarian cancer, histone acetylation promotes oncogene expression, while dysregulated histone deacetylase (HDAC) activity is implicated in ovarian and endometrial cancers, positioning HDAC inhibitors as promising therapies.^30^

Non‐coding RNAs (ncRNAs), particularly microRNAs (miRNAs), are emerging as key epigenetic regulators. Dysregulation of miRNAs like *miR‐200* and *miR‐34a* impacts cell proliferation, apoptosis, and metastasis in ovarian and endometrial cancers. For instance, *miR‐34a* downregulation enhances tumor growth and chemoresistance in ovarian cancer.^30^

## 4 IMPACT OF LIFESTYLE CHANGES ON SURVIVORS OF GYNECOLOGICAL CANCER

The survivorship of gynecological cancers is significantly influenced by lifestyle and environmental factors, with evidence suggesting that modifying risk factors after diagnosis can improve long‐term outcomes. Key lifestyle factors include smoking cessation, physical activity, diet, and maintaining a healthy body weight. A study on ovarian cancer survivors found that women who adopted a healthier lifestyle after their diagnosis, particularly those who quit smoking and increased physical activity, had better survival rates compared to those who did not make such changes. Specifically, women in the highest tertile of a healthy lifestyle index had a 39% lower risk of mortality compared to those in the lowest tertile.^31^

The American Cancer Society emphasizes the importance of nutrition and physical activity for cancer survivors, suggesting that these factors may improve survival and quality of life. For instance, higher physical activity after diagnosis was associated with a 40% reduction in mortality risk.^32^ In addition, a systematic review and meta‐analysis indicated that lifestyle interventions, including diet and physical activity, can positively impact overall survival and quality of life in survivors of gynecological cancer.^33^

Smoking cessation is particularly crucial, as current smoking after diagnosis is associated with significantly higher mortality. Women who quit smoking after diagnosis had survival outcomes similar to non‐smokers. Moreover, the adverse effect of smoking on survival may be stronger for women with *BRCA* variants compared to non‐carriers, highlighting the importance of targeted smoking cessation programs.^34^

Biological mechanisms underlying these associations include the impact of lifestyle factors on the tumor microenvironment and systemic inflammation. For example, physical activity has been shown to reduce systemic inflammation and improve immune function, which can enhance the body's ability to combat cancer cells.^35^ In addition, a healthy diet rich in fruits, vegetables, and whole grains can provide essential nutrients and antioxidants that support cellular repair and reduce oxidative stress.^35^

Lifestyle modifications have also been shown to reduce cancer recurrence rates. A meta‐analysis of randomized controlled trials found that lifestyle interventions significantly reduced the risk of cancer recurrence in gynecological cancer survivors.^33^ Furthermore, these interventions were associated with improvements in health‐related quality of life (HRQoL), including physical, emotional, and social well‐being.^33^, ^36^

Psychosocial factors also play a role in lifestyle changes after diagnosis. Cancer‐related concerns, such as health awareness and body change concerns, are associated with changes in physical activity and diet among gynecological cancer survivors.^37^ Addressing these psychosocial factors through tailored lifestyle advice and support can further enhance survivorship outcomes.^37^

## 5 DISCUSSION

The interplay between environmental exposures, lifestyle factors, and genetic predispositions is a key driver in the development of gynecological cancers.^1^, ^2^ Environmental factors, such as air pollution, industrial chemicals, and endocrine disruptors, contribute to oncogenesis by creating conditions that disrupt hormonal balance and immune function.^10^, ^13^ When coupled with modifiable lifestyle factors, including obesity, smoking, sedentary behavior, and poor dietary patterns, these exposures significantly amplify cancer risks.^7^, ^8^ Notably, periods of lockdown and remote work associated with the COVID‐19 pandemic have exacerbated these behaviors: reduced physical activity, increased stress‐related eating and mental disorders, and delayed routine screenings have collectively increased the incidence of cancer and likelihood of later‐stage diagnoses.^38^ While genetic predispositions, such as *BRCA* mutations and Lynch syndrome, play a central role in certain cancers, the broader impact of environmental and lifestyle determinants highlights the immense potential for preventive interventions, lying in the opportunity to mitigate risks through proactive public health strategies.

However, it is essential to acknowledge the social, economic, and cultural contexts that shape women's environments and lifestyles. Socioeconomic conditions directly influence exposure to environmental carcinogens, access to nutritious food, opportunities for physical activity, and healthcare services.^7^ Women from marginalized communities or lower socioeconomic strata often face disproportionate risks, exacerbated by limited access to education and preventive health care. These inequities not only increase cancer incidence but also delay diagnosis and reduce survival rates, creating a cycle of disparity that must be addressed.^7^

Globally, the burden of gynecological cancers reflects profound disparities driven by differences in healthcare infrastructure and societal factors. In high‐income countries, cancers such as endometrial and ovarian are frequently linked to lifestyle trends, including higher obesity rates, processed food consumption, and delayed childbearing.^7^ Urbanization and industrialization have also increased dependence on motorized transport, reducing daily physical activity, while simultaneously contributing to the proliferation of environmental pollutants that can trigger carcinogenic processes.^39^ Furthermore, the “westernization” of diets among migrant populations—where individuals shift from plant‐based, traditional diets to high‐fat, processed foods—has been associated with a heightened risk of cancer due to microbiome disruption, systemic inflammation, and hormonal imbalances.^39^ In contrast, cervical cancer remains predominant in low‐ and middle‐income countries (LMICs), where the lack of widespread HPV vaccination and organized screening programs limits opportunities for early detection and prevention.^7^ In fact, as highlighted in a study commissioned by the European Board & College of Obstetrics and Gynecology (EBCOG), 91% of countries have established cervical cancer screening programs. However, addressing the remaining disparities necessitates a coordinated effort to enhance healthcare accessibility, foster international collaboration, and implement culturally sensitive, community‐based interventions. Education and information dissemination are central to empowering women to make informed health decisions.^40^ Public health campaigns should emphasize the importance of healthy lifestyles, safe sexual behaviors, reproductive planning, and participation in regular cancer screenings and should begin as early as school age and continue through the onset of reproductive years. However, these educational strategies should be leveraged not only for the primary prevention of gynecological cancers but also to support gynecological cancer survivors, given the existence of several studies demonstrating the effectiveness of maintaining healthy lifestyles in reducing recurrence, increasing survival, and improving quality of life.^33^ Raising awareness about modifiable risks and advocating for systemic changes to reduce environmental exposures are equally important. HPV vaccination, for instance, represents a critical tool in reducing cervical cancer incidence, but its success depends on equitable implementation and widespread acceptance, particularly in underserved populations.^41^ Thus, it is important to highlight that several countries have developed educational programs, particularly targeting low‐income and low‐education populations, showing a significant increase and improvement in cervical cancer screening rates.^40^ Furthermore, at both national and international levels, policy measures aimed at reducing industrial pollution and occupational exposure to carcinogens, specific for female cancers, are equally crucial in diminishing environmental risks. Including such multifaceted strategies in national cancer prevention programs can also prove cost‐effective over time, as they reduce the long‐term burden on healthcare systems.

The evolving role of women in modern society further complicates the risk landscape. Increasing workforce participation, delayed motherhood, and urbanization have contributed to shifting reproductive and lifestyle patterns. These changes, while reflective of progress in some respects, have inadvertently increased hormonal and behavioral risk factors for certain cancers. Recognizing these societal trends is vital to developing gender‐specific prevention strategies that address the unique needs of women in different contexts.^3^

In this context, the integration of personalized medicine into cancer prevention and care provides an opportunity to address the complex interplay of genetic, environmental, and lifestyle factors. By tailoring interventions to individual risk profiles, personalized approaches can optimize prevention, early detection, and treatment. For example, women with hereditary cancer syndromes can benefit from enhanced surveillance, while population‐wide efforts targeting obesity, smoking cessation, and dietary improvements address broader risks. A clear example is represented by tools such as risk prediction models, including both modifiable and non‐modifiable risk factors (e.g. the CanRisk model), which offer promising avenues for identifying high‐risk individuals who may benefit from targeted preventive programs, although further research is needed to establish their clinical efficacy and feasibility in clinical practice.^42^ Integrating these approaches effectively will require robust healthcare infrastructures, as well as guidelines that balance innovation with equitable access and respect for patient autonomy. Promising preventive interventions, together with lifestyle modifications, can be implemented in high‐risk individuals, including prophylactic surgical removal of the reproductive organs in post‐menopausal women and bariatric surgery.^43^, ^44^ Indeed, among those at elevated risk for obesity‐related malignancies, it has been shown that bariatric surgery can induce substantial and sustained weight loss, which is linked to a lower overall cancer incidence and improved survivorship outcomes, particularly for endometrial and ovarian cancers.^44^, ^45^ Importantly, these strategies must be inclusive, ensuring that advances in personalized care are accessible to all women, regardless of socioeconomic status or geographic location.^46^

A gender‐sensitive approach to health care is critical to reducing disparities and improving outcomes for gynecological cancers. Public health systems must prioritize the integration of gender medicine into policy and practice, recognizing the distinct biological and sociocultural factors that shape women's health. Moreover, equitable access to preventive measures, from screening programs to vaccination, is essential for addressing disparities on a global scale. By combining personalized medicine with community‐based initiatives and international collaboration, it is possible to develop comprehensive solutions that empower women and reduce the burden of gynecological cancers worldwide.

## 6 CONCLUSION

The multifaceted nature of gynecological cancer prevention demands an integrated and comprehensive approach. By addressing environmental and lifestyle determinants, promoting equitable access to preventive measures, and adopting personalized and gender‐sensitive health care, significant progress can be made in reducing the global burden of these malignancies. Achieving this requires a commitment from healthcare systems, policymakers, and communities to prioritize women's health and ensure that every woman, regardless of her background, has the opportunity to benefit from advancements in prevention and care.

## AUTHOR CONTRIBUTIONS

Sara Farina and Alessandra Sabatelli contributed to the acquisition and interpretation of the data, and drafting the work. Stefania Boccia and Giovanni Scambia contributed to the conception of the work and interpretation of the data for the work, and reviewed critically. All the authors approved the final version to be published.

## CONFLICT OF INTEREST STATEMENT

The authors have no conflicts of interest.

## Data Availability

Data sharing is not applicable to this article as no new data were created or analyzed in this study.
